# Multiway real-time PCR gene expression profiling in yeast *Saccharomyces cerevisiae *reveals altered transcriptional response of *ADH*-genes to glucose stimuli

**DOI:** 10.1186/1471-2164-9-170

**Published:** 2008-04-16

**Authors:** Anders Ståhlberg, Karin Elbing, José Manuel Andrade-Garda, Björn Sjögreen, Amin Forootan, Mikael Kubista

**Affiliations:** 1 TATAA Biocenter, Odinsgatan 28, 411 03 Göteborg, Sweden; 2 Stem Cell Center, Lund University, BMC B10, 221 84 Lund, Sweden; 3 Deparment of Cell and Molecular Biology, Göteborg University, BOX 462, 405 30 Göteborg, Sweden; 4 Department of Analytical Chemistry, University of A Coruna, A Zapateira s/n, E-15071 A Coruna, Spain; 5 Center for Applied Scientific Computing, Lawrence Livermore National Laboratory, Box 808, L-365, Livermore, CA 94551, USA; 6 MultiD Analyses AB, Odinsgatan 28, 411 03 Göteborg, Sweden; 7 Institute of Biotechnology, Academy of Sciences of the Czech Republic, Videnska 1083, Prague 4, 142 20, Czech Republic; 8 Department of Clinical Neuroscience and Rehabilitation, Institute of Neurosciences and Physiology, Sahlgrenska Academy at Göteborg University, Medicinaregatan 9A, 413 90 Göteborg, Sweden

## Abstract

**Background:**

The large sensitivity, high reproducibility and essentially unlimited dynamic range of real-time PCR to measure gene expression in complex samples provides the opportunity for powerful multivariate and multiway studies of biological phenomena. In multiway studies samples are characterized by their expression profiles to monitor changes over time, effect of treatment, drug dosage etc. Here we perform a multiway study of the temporal response of four yeast *Saccharomyces cerevisiae *strains with different glucose uptake rates upon altered metabolic conditions.

**Results:**

We measured the expression of 18 genes as function of time after addition of glucose to four strains of yeast grown in ethanol. The data are analyzed by matrix-augmented PCA, which is a generalization of PCA for 3-way data, and the results are confirmed by hierarchical clustering and clustering by Kohonen self-organizing map. Our approach identifies gene groups that respond similarly to the change of nutrient, and genes that behave differently in mutant strains. Of particular interest is our finding that *ADH4 *and *ADH6 *show a behavior typical of glucose-induced genes, while *ADH3 *and *ADH5 *are repressed after glucose addition.

**Conclusion:**

Multiway real-time PCR gene expression profiling is a powerful technique which can be utilized to characterize functions of new genes by, for example, comparing their temporal response after perturbation in different genetic variants of the studied subject. The technique also identifies genes that show perturbed expression in specific strains.

## Background

The extraordinary sensitivity and virtually unlimited dynamic range of real-time PCR makes it the preferred technology for quantitative gene expression profiling. Using microarray technology expression of entire genomes can be measured, identifying candidates for expression profiling. After validation of these genes on representative samples by real-time PCR, eliminating any false leads and possibly complementing with other genes, powerful panels of expression markers can be identified. The recent development of high throughput real-time PCR platforms [1,2] will spur the development further. To extract maximum information from profiling experiments using such panels, methods to pre-process and process the gene expression data are needed.

The addition of glucose to *Saccharomyces cerevisiae *cells grown in ethanol causes an extensive reprogramming of gene expression and metabolism, making it a suitable model system to understand gene regulation. In this system glucose consumption rate correlates with glucose repression [3,4]. We have previously reported on a series of strains, in which glucose uptake is mediated by different native and chimeric hexose transporters, which display a wide range of glucose uptake rates [3,5,6]. These strains are useful for investigating the effects of different glycolytic rates on glucose-induced signaling pathways. Many glucose induced and glucose repressed genes have been extensively studied, but several genes believed to be associated with metabolism remain poorly characterized. The alcohol dehydrogenases (*ADH*) are such group. Its first two members, *ADH1 *and *ADH2*, have well known characteristics [7-10], while the functions of *ADH3-6 *are poorly understood [11-16].

We have previously shown that glucose uptake is the rate limiting step for glycolytic flux in strains expressing a series of individual glucose transporters with reduced transport capacity [5]. Ethanol production, also under aerobic conditions, in *Saccharomyces cerevisiae *is believed to be a result of overflow metabolism where rate limitation of the TCA cycle results in a flux towards ethanol production [17]. In this study we were interested in exploring transcriptional responses of some of the less characterized *ADH*-genes to better understand their regulations under conditions of different glycolytic rates.

Here, four yeast strains representing the full range of glycolytic rates; namely, *wild-type *(high glycolytic rate), *HXT-HXT7 *(medium glycolytic rate), HXT-TM6* (low glycolytic rate), and *HXT-null *(no glucose uptake) were selected to study the responses of metabolic genes. In previous works we have shown that ethanol production rate correlates to glycolytic rate [3], which pointed us in the direction of the *ADH*-genes and in particular the less studied *ADH3*, *ADH4*, *ADH5 *and *ADH6*. Here we address whether a decrease in the rate of ethanol production rate can be attributed in part to the roles of the *ADH*-genes. The study is a 3-way design, with the three ways being (i) genotype, (ii) gene, and (iii) time. We use a variant of geNorm [18] and Normfinder [19] to find suitable reference genes for normalization along all three ways, and we present suitable pre-processing of the data for analysis. Finally, the data are analyzed by augmented principal component analysis (PCA), which is a variant of PCA for 3-way studies [20]. The classification obtained by the augmented PCA is verified by hierarchical clustering and clustering by the self-organizing map (SOM) [21]. The analyses classify the genes into five groups with characteristic temporal profiles, based on which functional similarities between the *ADH3-6 *genes and previously more characterized genes can be found.

## Results and Discussion

### Experimental setup and gene selection

In this study four yeast strains KOY.PK2-1C83 (*wild-type*), KOY.HXT7P (*HXT-HXT7*), KOY.TM6*P (HXT-TM6*) and KOY.VW100P (*HXT-null*) were used. In the *HXT-HXT7*, HXT-TM6* and *HXT-n*ull strains, the genes *HXT1-HXT7*, *GAL2*, *STL1 *and three maltose permeases (see Methods) have been deleted. This results in a strain unable to take up glucose as measured by uptake of radiolabeled C^13^-glucose [22]. Into this *HXT-null *strain we introduced either *HXT7 *(*HXT-HXT7*) or TM6* (HXT-TM6*). The four strains used have high, intermediate, low and zero glucose consumption rates, *i.e. *glycolytic rates [3,5,6]. They were grown on ethanol and exposed to glucose to follow the transcriptional responses of a series of selected genes. Samples were collected before and during one hour after the pulse to study the short term response of the genes.

**Figure 1:**
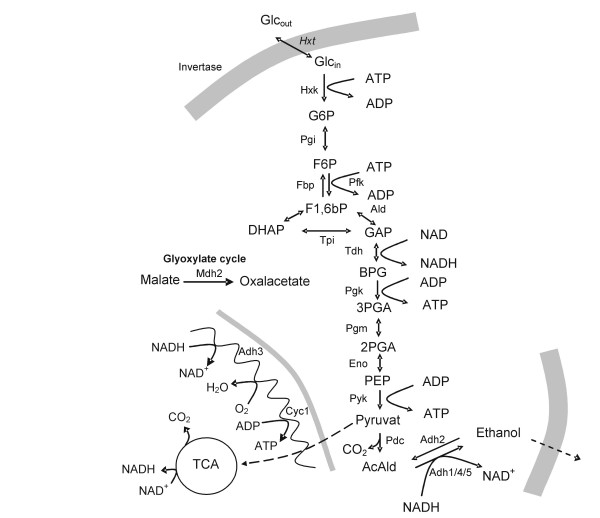
Schematic diagram of central metabolism to indicate position of relevant/studied enzymes in metabolism.

Genes known to be induced or repressed in *wild-type *strain, with normal glycolytic flux, were included in the study to allow for the classification of the *ADH*-genes (Figure 1). The established glucose induced genes were Triose-phosphate isomerase 1 (*TPI1*, catalyzing reaction step 5), Phosphoglycerate kinase 1 (*PGK1*, catalyzing reaction step 7), Pyruvate decarboxylase (*PDC1*, catalyzing reaction step 11) and Alcohol dehydrogenase 1 (*ADH1*, catalyzing reaction step 12), which are all members of the lower glycolysis [8,23]. The established glucose repressed genes involved in gluconeogenesis and the glyoxylate cycle genes were Fructose-1,6-bisphosphatase (*FBP1*, catalyzing the reverse reaction of step 4), Alcohol dehydrogenase 2 (*ADH2*, catalyzing the reverse reaction of step 12) and Malate dehydrogenase 2 (*MDH2*, catalyzing oxalacetate in the glyoxylate cycle) [23]. In addition, Sucrose fermentation (*SUC2*) was included, which is a well studied target gene of glucose repression/derepression [24-27], the Multicopy Inhibitor of *GAL *(*MIG1*), which is a central repressor of *SUC2 *[28], the mitochondria localized Cytocrome C (*CYC1*), which is regulated by *MIG1 *and repressed by glucose [29,30] and heat shock protein 12 (*HSP12*), which is known to be repressed by very low glucose concentrations and is also stress induced [31-33]. Inorganic pyrophosphatase (*IPP1*), Actin (*ACT1*), and Pyruvate dehydrogenase 1 (*PDA1*) were included as tentative house-keeping/reference genes. In addition to the well characterized genes, for which raw data have been partly reported before [3], we also included a series of *ADH*-genes (*ADH3-6*) that are less well understood, to investigate their responses in relation to different glycolytic rates. Throughout this paper the genes in figures and tables are color coded as follows: *ADH1*, *PGK1*, *TPI1*, *PDC1 *and *MIG1 *genes, which are expected to be induced by glucose, are shown in blue, *FBP1*, *ADH2*, *MDH2 *and *SUC2*, which are expected to be repressed by glucose, are shown in red, *ADH3*, *ADH4*, *ADH5 *and *ADH6 *genes, whose functions are rather unknown, are shown in yellow, *HSP12 *is shown in black and *CYC1 *in green.

### Validation of reference genes

**Figure 2:**
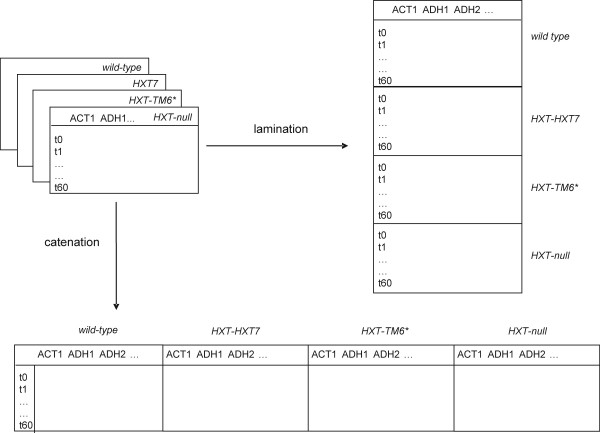
Diagram showing lamination and catenation of data matrices.

**Figure 3:**
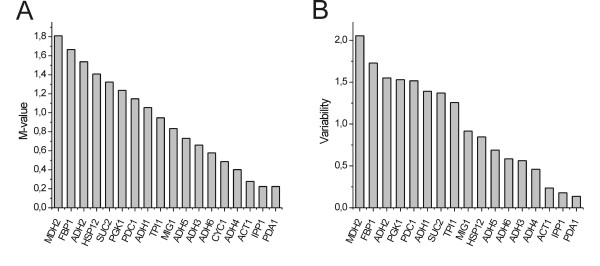
**Identification of potential reference genes.** Potential reference genes are identified by (A) geNorm and (B) NormFinder. Low M-value or variability represents the most constantly expressed reference genes.

For proper comparison samples should be normalized. Parameters such as mass, volume, cell number and total RNA have been used but none of these can compensate for variations in RNA quality and the presence of reaction inhibitors. Today, normalization is usually performed with internal reference genes, which always should be validated to have constant expression under the conditions of the study [34]. Identifying appropriate reference genes for data normalization and validating them on representative samples is a challenging problem in expression profiling, because the expression of all genes seem to be regulated under some conditions. Different algorithms have been developed to identify the most suitable reference genes. geNorm [18] and NormFinder [19] are among the most popular. The two methods are based on somewhat different assumptions. While geNorm identifies the pair of genes with most correlated expression relative to all the other genes by an elimination approach, Normfinder identifies the gene(-s) that shows least variation. Normfinder also distinguishes between intra- and inter-group variation, where the latter is the systematic difference in expression between the subgroups (here the four strains). The data, in the form of cycle of threshold (CT)-values, were arranged in one matrix per strain, with the genes as columns and the sampled time points as rows. The two PCR replicates measured for each gene were averaged. The four 8 × 18 matrices were then laminated into a 32 × 18 matrix (Figure 2) and an extra classification column was added to index the strains. The results of geNorm and Normfinder are shown in Figure 3. geNorm, which treats all data as a homogenous group, identified *PDA1 *and *IPP1 *as the most stable pair of genes and *ACT1 *as the 3rd best reference gene candidate. Normfinder selected the genes in the stability order *PDA1 *> *IPP1 *> *ACT1*, with all exhibiting insignificant intra and intergroup variability. Hence, we conclude that *ACT1*, *IPP1 *and *PDA1 *are suitable reference genes for our study of yeast metabolism. This conclusion was supported by principal component analysis, which showed that *ACT1*, *IPP1 *and *PDA1 *cluster in scatter plots evidencing that they have similar behavior (data not shown).

### Data pre-processing

Data pre-processing was performed starting with the four matrices containing the measured CT-values. All CT-values reflected formation of targeted product as verified by melting curve analysis, and corrections for primer-dimer signals were not needed. For strain HXT-TM6* both technical repeats had failed for *PDA1 *at 30 min, and the missing data was reproduced by interpolation. The CT-values were corrected for PCR efficiency (E) assuming E = 0.95 for all assays in all strains:

$$CT_{E=100\%}=CT_{E}\frac{\log\left(1+E\right)}{\log\left(2\right)}$$

95% PCR efficiency was typical for the assays used in the *Saccharomyces cerevisiae *matrix. However, it should also be stressed that correction for PCR efficiency has negligible effect on classification of multivariate expression data [19]. The PCR technical repeats were then averaged. Next the CT-values for the genes of interest (GOI) were normalized with the average of the CT-values of the reference genes (RG) *PDA1*, *IPP1 *and *ACT1*:

$$CT_{GOI,norm}=CT_{GOI}-\frac{1}{n}\sum_{i=1}^{n}CT_{RG}$$

This reduced the dimension of the data matrices to 8 × 15. Next, the CT-values were converted to relative quantities (RQ)

$$RQ=2^{CT_{0}-CT}$$

where CT_0 _is the CT-value measured immediately before glucose addition (0 minutes). RQ was then converted to fold changes (FC) with log_2 _base:

*FC *= log_2_(*RQ*)

**Figure 4:**
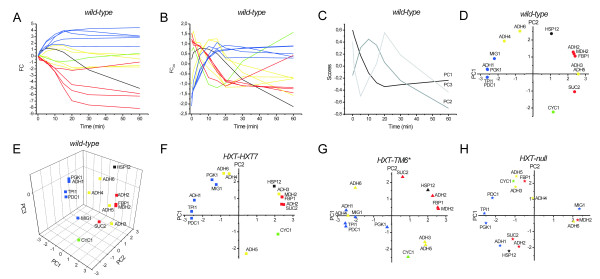
**Classification of genes using PCA.** The FC (A) and FC_AS_(B) over time are shown for *wild-type *yeast. Using PCA the first three scores vectors (C) are shown together with PC1-PC2 (D) and PC1-PC2-PC3 (E) plots for *wild-type *yeast. Corresponding PC1-PC2 plots are shown for the *HXT-HXT7 *(F), HXT-TM6* (G), and *HXT-null *(H) strains. The following colors and symbols are used: Glucose-induced genes (blue), glucose-repressed genes (red), *ADH3-6 *(yellow), *HSP12 *(black), *CYC1 *(green), *wild-type *(circles), *HXT-HXT7 *(squares), HXT-TM6* (triangles) and *HXT-null *(stars).

The temporal expression profiles, expressed as FC, are shown for the *wild-type *strain in Figure 4A. To give all genes equal weights for classification of expression profiles the data were autoscaled (FC_AS_) by subtracting the mean expression of every gene in each strain (i.e, the column mean) and dividing with the (column) standard deviation:

$$FC_{AS}=\left(FC-\bar{FC}\right)/SD$$

The average expression of each gene in each strain is now zero and its standard deviation is one. The autoscaled expression profiles for the yeast wild-type strain are shown in Figure 4B (RQ and FC_AS _for the other strains are shown in Additional data 1).

### Classification of the genes' expression profiles with principal component analysis

The measured data consisted of eight time points (samplings) measured for fifteen genes of interest in each of the four strains. Hence, the total number of data points was: 8 (samplings) × 15 (genes) × 4 (strains) = 480. Clearly, to visualize such large amounts of data and to unravel patterns efficient methods are called for. The classical scatter plot is an intuitive way to visualize how genes are expressed in different samples. These are typically 2-dimensional plots where the two axes indicate the genes' expressions in the two samples. It is possible to indicate genes' expressions for three genes in a 3-dimensional scatter plot. But this is the limit, since we have no convenient way to plot data in more than three dimensions. To deal with higher order data multivariate biostatistical tools are required to reduce the number of dimensions without loss of essential information. A classical, widely used tool is Principal Components Analysis (PCA). PCA allows scientists to study many variables simultaneously. It reflects how the original variables are correlated, and also how the samples are grouped. Principal Components (PCs) are mathematical constructs that can be interpreted as linear combinations of the studied variables with the following important properties:

(i) The PCs are orthogonal. Once a PC is linked to the behavior of one or several genes, one can be reasonably sure that this correlation is unique and these genes do not correlate substantially with other PCs. The numerical coefficients, ranging from -1 to +1, given to each gene in each PC are called loadings and reflect how important the gene is to define this PC.

(ii) The PCs are sequential. This means that the first, and by definition the most significant, PC can be interpreted as the line in the original multidimensional space of all the samples that best fits the expression data and, hence, explains most of the observed variability and accounts for most of the information. The second most significant PC can be visualized as a vector perpendicular to the first PC that fits the expression data best, and accounts for most of the variability that is not accounted for by the first PC. Additional PCs are defined analogously. One can extract PCs until a certain percentage of all the information, let's say 80%, is accounted for, and then discard the remaining PCs, which will mainly reflect uncorrelated information and, hence, the experimental noise. In most cases it is not practical to calculate more than three PC's, since, as already mentioned, we are limited to make 3-dimensional plots. Once the PCs have been calculated, the samples can be located in this new space using the scores, which specify the location of each sample on each PC. The original data can now be presented in a scores scatter plot to reveal groups among the samples or a loadings scatter plot to reveal groups among the genes. Many times PC1-PC2 scatter plots are sufficient, but one can also construct PC1-PC2-PC3 scatter plots.

Figure 4C shows the two most significant scores vectors for the autoscaled *wild-type *(high glycolytic rate) yeast data. As the temporal expression profiles, the scores are functions of time, and they reflect the main features of all the measured profiles. The most significant PC score vector (PC1) reflects a rapid decrease in expression that levels off after some 20 minutes. The PC2 score vector is signifying extreme expression at an intermediate time of about 10–15 minutes. The expression profile of every gene can be approximated as a linear combination of the two scores vectors, and can be visualized in a scatter plot based on the weights (loadings) of the linear combination (Figure 4D). For wild-type yeast we see that the induced (blue) and repressed (red) genes are clearly distinguished by PC1: induced genes have negative PC1 loadings while repressed genes have positive. Among the repressed genes, we find that *SUC2 *is located off the cluster's center, indicating that the *SUC2 *profile may be somewhat different from that of the bulk of the repressed genes. In Figures 4A and 4B, we indeed see that one of the red temporal expression profiles shows a different behavior from the rest. This is *SUC2*. While the expression of the bulk of the repressed genes reaches minimum at about 20 minutes and then saturates, *SUC2 *expression goes through a minimum and thereafter it slowly increases. Indeed, the PC2 loadings sort genes based on their tendency to show extreme expression at an intermediate time of about 10–15 minutes (the PC2 scores vector in Figure 4C). Genes showing local maximum expression at an intermediate time are characterized by a positive PC2 loading, while genes exhibiting local minimum have negative PC2 loading. Genes that do not show extreme expression at intermediate time points have PC2 loadings around zero. The most negative PC2 loading is found for *CYC1 *(figure 4D), which shows maximal repression after about 15 minutes after glucose addition (Figure 4B). Positive PC2 loadings are found for *HSP12*, *ADH4 *and *ADH6*, which all have a local maximum in their temporal expression profile. The specific roles for Adh4 and Adh6 are not fully understood. *ADH4 *has previously been reported not to be expressed in laboratory strains or to affect ethanol production [35]. *ADH6 *has a high specificity towards long chain aliphatic and bulky substrates and has been suggested to participate in the production of fusel alcohols [12]. Fusel alcohols are produced mostly during fermentation, which could explain its induction after glucose addition. The induced genes, with negative PC1 loadings, and most of the repressed genes, with positive PC1 loadings (*SUC2 *being an exception), have PC2 loadings close to zero, indicating that their temporal expression profiles are unimodal. *ADH3 *and *ADH5 *are located close to each other in the loadings scatter plot, within the cluster of the repressed genes. They have somewhat lower PC2 loadings than the bulk of the repressed genes, but higher than *SUC2 *(Figure 4D). This suggests that *ADH3 *and *ADH5 *respond similarly to glucose addition in *wild-type *yeast, and their expression profiles are characteristic of repressed genes. Repression of *ADH3 *after glucose addition is consistent with previous studies, which report lower Adh3 activity during respiratory growth when compared to fermentative growth [14]. Adh5 has been reported to be involved in ethanol formation but its function is only apparent in an *adh1adh3 *strain [13]. More detailed studies are needed to understand differences in responses between *ADH4*/*6 *and *ADH3*/*5*.

From the eigenvalues of the PCA it was calculated that 95.6 % of the variance in all the measured data is accounted for by the first two PC's, reinforcing the usefulness of the PC1 vs. PC2 loadings scatter plot. The third PC of the *wild-type *strain accounts for an additional 3% of the variability in the data. The third score vector has the shape of the derivative of the second vector (Figure 4C), and sorts the genes based on when they reach intermediate extreme expression. Genes that reach extreme expression after 10–15 minutes obtain positive PC3 loading, while genes reaching extreme expression within 10 minutes obtain negative PC3 loading. The genes in *wild-type *yeast are clustered based on all three PC's in the PC1 vs. PC2 vs. PC3 loadings scatter plot in Figure 4E. In the 3D loadings scatter plot, *MIG1 *separates from the other induced genes, because its expression reaches maximum at an earlier time. The 3-dimensional plot accounts for 98.6 % of all the variability, which is essentially all biologically relevant information; remaining variability is mainly experimental noise. This can be verified by comparing the *wild-type *temporal expression profiles with those reproduced from the three main PC's. The agreement is excellent evidencing that the three PC's have indeed picked up all the important features of the genes' expression profiles in *wild-type *yeast (data not shown). These features are:

1) Expression either increases or decreases

2) Expression reaches an extreme negative or positive level at an intermediate time point from which it recovers

3) Extreme expression is reached before or after 10 minutes.

PCs calculated for *HXT-HXT7 *(medium glycolytic rate) had similar features as those for the *wild-type *strain. In the PC1 vs. PC2 loadings scatter plot (92.6% of the initial variance, Figure 4F), the PC1 still differentiates between induced (left) and down-regulated (right) genes. The repressed genes cluster more tightly in the *HXT-HXT7 *strain compared to *wild-type*, and the cluster also contains *ADH3 *and *HSP12*, which in *wild-type *had a distinct location. This is because their expression in *wild-type *initially increases, goes through a maximum (Figure 4A–B) and then decreases. In *HXT-HXT7 *they are instead repressed from start. *ADH5*, which was found close to the cluster of repressed genes in *wild-type*, is now distant from it having large negative PC2 loading. This suggests that its expression is affected in *HXT-HXT7 *such that it goes through a minimum. *CYC1*, which too has negative PC2 score, also goes through a minimum in its temporal expression profile in the *HXT-HXT7 *strain. In addition, the induced genes separate into two subclusters: *ADH1*, *TPI1 *and *PDC1 *in one and *PGK1*, *MIG1*, *ADH4 *and *ADH6 *in the other (Figure 4F). The separation is mainly along PC2, indicating that the subgroups differ in whether expression goes through (in this case) a maximum or if it levels off.

For HXT-TM6* (low glycolytic rate)*the *contribution from noise to the data was larger due to the overall less efficient glucose response and the two main PC's account for only 85 % of the total variance. PC1 loadings still reflect whether genes are up or down-regulated, while the PC2 loadings are less well defined (Figure 4G). The repressed genes still form a cluster, with *SUC2 *having a somewhat higher PC2 score. *HSP12 *is located within the cluster of repressed genes, while *ADH3 *and *ADH5*, which were within this cluster in *wild-type *strain, are separated due to negative PC2 loadings and they are now closer to *CYC1*. The induced genes, but *PGK1*, form a tight cluster containing also *ADH4*. *ADH6 *is found at similar PC1 score as the induced genes, suggesting it too has similar expression, although its positive PC2 score indicates its expression profile is somewhat shifted in time. *PGK1 *is located around zero loadings for both PC1 and PC2. This means that the first two PCs are not very useful to describe its temporal profile and we can only conclude it is different from that of the other induced genes. Inspecting the raw data (see Additional data 1) we find that *PGK1 *expression in HXT-TM6* is essentially unaffected by glucose addition and remains at a constant level.

For the *HXT-null *(no glucose uptake) strain the changes in expression upon glucose addition were small, and the PC loadings are quite different from those of the other strains. Therefore, the genes' locations in the *HXT-null *scatter plot cannot be compared to the previous ones. Anyway not much remains of the groups seen for the other strains, reflecting the relatively weak response in *HXT-null *when glucose is added. The most spectacular observation is that *MIG1*, which is a typical glucose-induced gene, is here clearly differentiated from the other glucose induced genes (Figure 4H and Additional data 1C). Inspection of the temporal profiles indicates that *MIG1 *is the only gene in this group that is still induced upon glucose addition; the expression of the other genes decreases when glucose is added. *HSP12*, which is repressed after the glucose pulse has previously been reported to be down-regulated at very low glucose concentrations [33], but our results rather suggest that the signal is extracellular. Our observation that *MIG1 *is still derepressed in *HXT-null *confirms that it is regulated through extracellular sensing, which previously has been suggested by Kaniak *et al*. [36].

**Figure 5:**
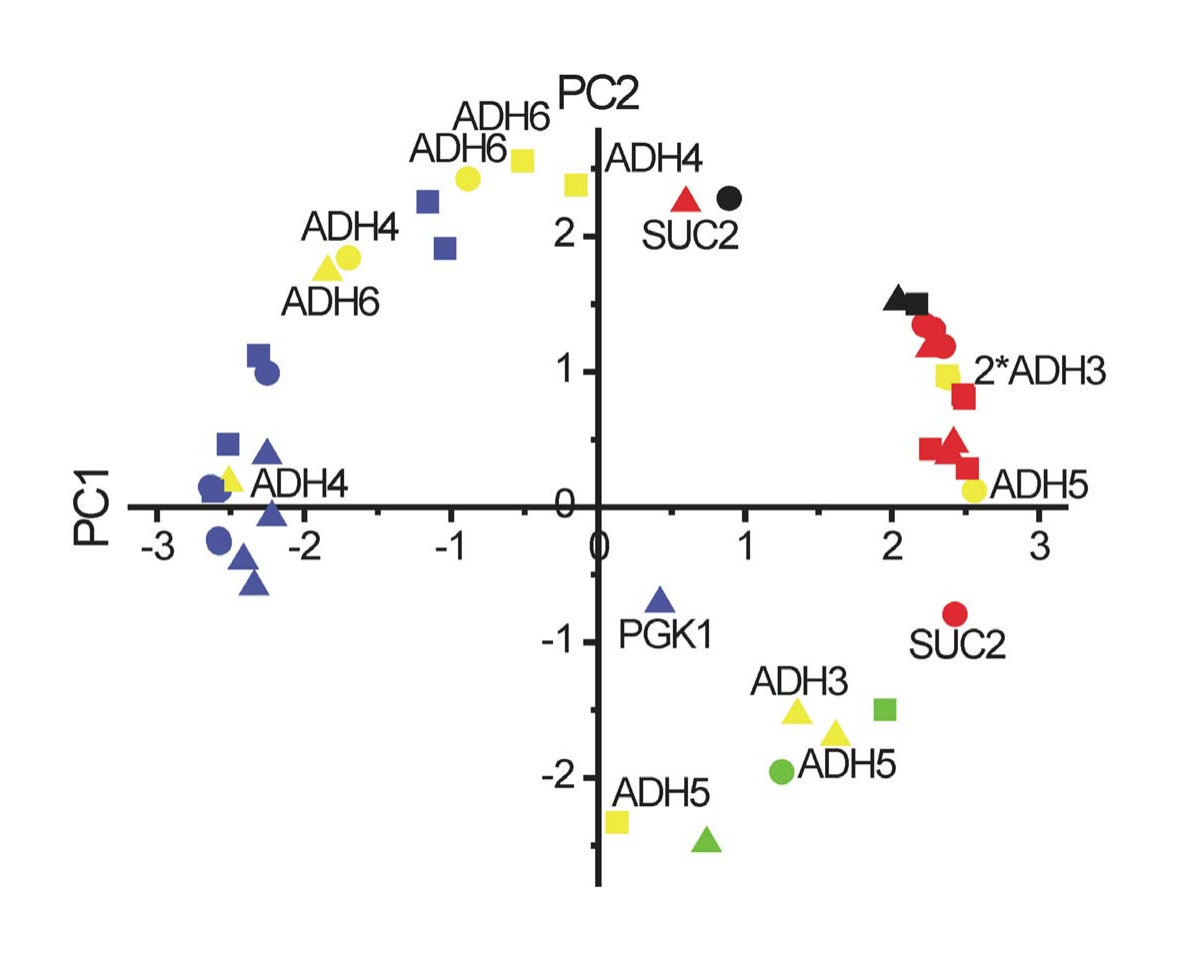
Matrix-augmented PCA for *wild-type*, *HXT-HXT7 *and HXT-TM6* yeast. Data matrices from the respective strains were catenated to a single matrix, followed by PCA. The following colors and symbols are used: Glucose-induced genes (blue), glucose-repressed genes (red), *ADH3-6 *(yellow), *HSP12 *(black), *CYC1 *(green), *wild-type *(circles), *HXT-HXT7 *(squares) and HXT-TM6* (triangles).

In traditional PCA (as performed above) the PC's are calculated for each strain separately, yielding strain specific score and loading vectors. As consequence, the loadings plots obtained for the different strains cannot be compared easily because the axes have different orientations in the original multidimensional measurement space. This problem is evident when comparing the *HXT-null *above with any of the other strains: for all the other strains genes induced upon glucose addition are characterized by negative PC1 loadings, while for *HXT-null *induced genes have positive PC1 loadings. The reason is that the PC1 loading vectors have (among other things) opposite orientations in the two cases. To deal with this problem the study must be treated as multiway. While multivariate methods are designed to study one set of samples, characterized by the response of many variables (= genes), multiway methods should be used to study several sets of samples, such as the four strains here. One multiway method is matrix-augmented PCA [37]. In matrix-augmented PCA the pre-processed data matrices can be either laminated or catenated (Figure 2). Since we here are primarily interested in the genes we catenate the data into an 8 × 60 matrix, with the genes as columns and samplings as rows. PCA is then performed on the augmented matrix, which produces common score vectors for all strains. These were used to construct the PC1 vs. PC2 loadings scatter plot in Figure 5. In the plot, the genes are color coded as before and the four strains are distinguished by symbols. The *HXT-null *strain was omitted for clarity. A plot containing also *HXT-null *is provided in Additional data 2. The two main PC's account for 87% of the variability in the catenated data set and the loading vectors have shapes similar to those of the *wild-type *strain shown in Figure 4D. Five main areas with genes are seen.

I. PC1 << 0, PC2 ≈ 0; genes induced upon glucose addition.

II. PC1 >> 0, PC2 ≈ 0; genes repressed upon glucose addition.

III. PC1 ≈ 0; PC2 >> 2; genes with expression profiles that go through a maximum.

IV. PC1 ≈ 0; PC2 << 2; genes with expression profiles that go through a minimum.

V. PC1 ≈ 0; PC2 ≈ 0; genes with no regulation upon glucose addition.

Most of the *ADH1*, *PGK1*, *TPI1*, *PDC1 *and *MIG1 *genes are found in area I, indicating they are induced upon glucose addition. Exceptions are *PGK1 *and *MIG1 *in the *HXT-HXT7 *strain, which go through a maximum, and *PGK1 *in HXT-TM6*, which shows no response. Most *FBP1*, *ADH2*, *MDH2 *and *SUC2 *genes are in area II. Exceptions are *SUC2 *in *wild-type*, where it shows a local minimum in the temporal profile, and in HXT-TM6*, where it shows a transient induction similar to that observed in growing cells close to glucose depletion [24]. The less well understood *ADH3-6 *genes show the following behavior: *ADH4 *and *ADH6 *are found in areas I and III co-localized with genes induced by glucose, while *ADH3 *and *ADH5 *are found in areas II and IV co-localized with genes repressed by glucose. Hence, we conclude this is their general response to glucose. Several of the *ADH3-6 *genes in some strains have PC2 scores significantly different from zero indicating that their temporal profiles may go through local minima/maxima. To verify these conclusions the entire experiment was repeated and analyzed separately by matrix-augmented PCA (Additional data 3). Only small differences in scores were seen and the genes were grouped the same way as in Figure 5

### Confirmation with hierarchical clustering and Kohonen self-organizing maps

While PCA is a very robust approach to classify samples based on multivariate and multiway measurements and an excellent tool to unravel variable patterns, there are also other techniques for unsupervised clustering. The most common is hierarchical clustering although, recently, the Kohonen self-organizing map (SOM) is gaining attention. While PCA always yields the same unbiased result for a certain set of data, hierarchical clustering requires the user to select a distance or similarity measure and also to define how distances between groups shall be measured. Once these decisions are made, hierarchical clustering also gives the same result every time for certain data. SOM, on the other hand, is based on a particular type of artificial neural networks that can be used to create an organized map of expression profiles by treating the raw information in the experimental data using a chain of successive comparisons. The goal is to create a map where adjacent areas correspond to similar samples, alternatively to genes with similar expressions. A SOM consists of an array of unconnected artificial neurons. There are several options to organize them but in most cases they are arranged in a convenient square so that each neuron is adjacent to other neurons (termed neighbors). The underlying idea is to assign sets of neurons (regions in the map) to a distinct class of samples. This is achieved through an iterative process. Briefly, each neuron in the map is defined as a set of weights (a vector of values) that equals the number of variables, in this case temporal profiles, measured for each gene [38]. Hence, the weight vectors can be interpreted as artificial expression profiles. Creating the SOM consists of adjusting the weights of the neuron during a training phase. First the map is initiated by assigning small random numbers to the weights. Then a gene is selected randomly and its measured temporal expression profile is compared to the weights of the SOM's neurons. The neuron with the most similar weights is identified and information about the gene's expression profile is added to its weights. Furthermore, the information is also added to the neighboring neurons. The process is then repeated with a new gene over and over until a stable SOM is obtained. This SOM will have neurons with weights that reflect all genes' expression profiles and weights of neighboring neurons will be similar. In a final step each gene is placed in the neuron of the SOM that has the most similar weight to its temporal profile. As a consequence genes with similar profiles will be found in the same neuron or in close-lying neurons.

The possible advantages of hierarchical clustering and SOM when compared to PCA are that the former methods are intuitive and use all the information in the data, while PCA is based on coordinate transformation and reduction of dimensionality, which are less obvious operations and also always loses some of the variability in the measured data. The loss of limited amount of information is usually not serious, at least if the first 2–3 PCs have large eigenvalues and, hence, account for a large percentage of the total variation. In fact, reduction of dimensionality may lead to improved signal to noise ratio in the measured data, since biological variability is systematic and is mainly contained in the initial PCs, while experimental noise is random and dominates the PCs that are discarded [39]. A disadvantage of hierarchical and Kohonen-SOM clustering is that they only yield groups, and any further biochemical interpretation has to be extracted studying the original data matrices, while in PCA there is a relation between the measured data and the scores and loadings.

**Figure 6:**
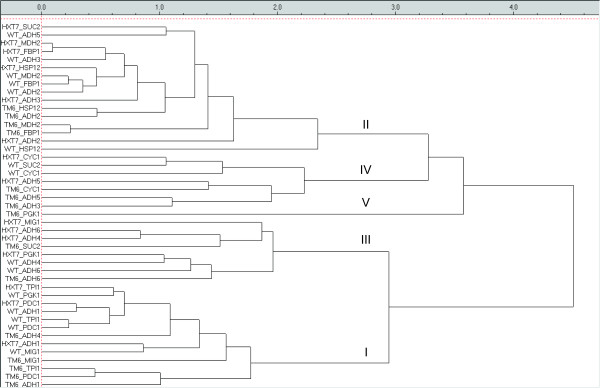
Hierarchical clustering of the genes in the *wild-type*, *HXT-HXT7 *and HXT-TM6* strains. Un-weighted pairs were used to calculate similarities between clusters based on Euclidian distances. Five different groups are identified and marked in the plot.

Figure 6 shows the graphical output (dendrogram) of the hierarchical clustering of the catenated *wild-type*, *HXT-HXT7 *and HXT-TM6* autoscaled data, using the Euclidean distance to measure similarity and the unweighted pairs clustering algorithm [40]. The dendrogram reveals four main groups: from top, the first group is composed of *MDH2*, *FBP1*, *HSP12 *and *ADH2 *from all three strains, *SUC2 *from *HXT-HXT7*, *ADH3 *from *wild*-*type *and *HXT-HXT7*, and *ADH5 *from *wild*-*type*. Group 2 contains *CYC1 *from all strains together with *SUC2 *from *wild-type *and *ADH5 *from HXT-TM6* and *HXT-HXT7 *and *ADH3 *from HXT-TM6*. Group 3 contains all *ADH6 *and *ADH4 *from *wild*-*type *and *HXT-HXT7*, and *PGK1 *and *MIG1 *from *HXT-HXT7*. Last group contains all *TPI1*, *ADH1*, and *PDC1*, together with *MIG1 *from *wild*-*type *and HXT-TM6*, PGK1 from the *wild*-*type *and *ADH4 *from HXT-TM6*. *PGK1 *in HXT-TM6* belongs to no group evidencing it has a unique response. The four groups as well as the unique location of *PGK1 *in HXT-TM6* agree well with the four regions in the PC1 vs. PC2 loadings scatter plot of the PCA shown above (Figure 5) reinforcing the previous conclusions.

**Figure 7:**
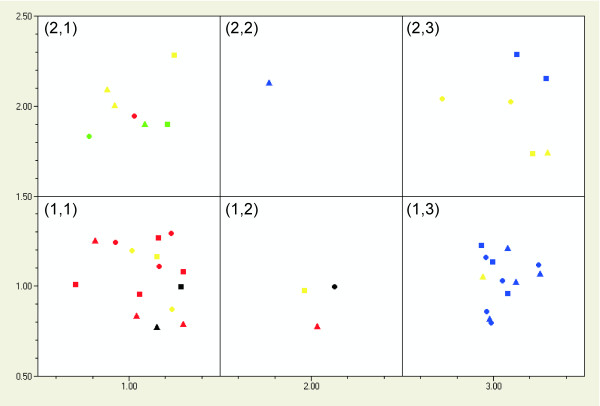
Kohonen self-organizing maps for the augmented *wild-type*, *HXT-HXT7 *and HXT-TM6* yeast. A map with six cells was used to force classification into six groups. A learning rate of 0.10, 2 neighbors and 10000 iterations were used. This gave the following groups: (1, 1) *FBP1*, *ADH2*, *MDH2 *(all strains), *SUC2 *(*HXT-HXT7*) *HSP12 *(*HXT-HXT7 *and HXT-TM6*), *ADH3 *(*wild-type *and *HXT-HXT7*) and *ADH5 *(*wild-type*); (1, 2) *SUC2 *(HXT-TM6*), *HSP12 *(*wild-type*) and *ADH4 *(*HXT-HXT7*); (1, 3) *ADH1*, *PDC1*, *TPI1 *(all strains), *MIG1 *(*wild-type *and *HXT-HXT7*), *PGK1 *(*wild-type*), *ADH4 *(HXT-TM6*) (2, 1) *CYC1 *(all strains), *SUC2 *(*wild-type*), *ADH5 *(*HXT-HXT7 *and HXT-TM6*), *ADH3 *(HXT-TM6*); (2, 2) *PGK1 *(HXT-TM6*); (2, 3) *MIG1 *(*HXT-HXT7*), *PGK1 *(*HXT-HXT7*), *ADH4 *(*wild-type*), *ADH6 *(all strains). The following colors and symbols are used: Glucose-induced genes (blue), glucose-repressed genes (red), *ADH3-6 *(yellow), *HSP12 *(black), *CYC1 *(green), *wild-type *(circles), *HXT-HXT7 *(squares) and HXT-TM6* (triangles).

There are two strategies to design SOM. A fairly large SOM, typically of dimension n × n, with n being the number of genes, can be used. In such SOM genes are sparse and only rarely is more than one gene found in one neuron. There are no clear boundaries between groups, because the SOM surface changes irregularly and distances between the genes are not proportional to the differences between them. Still, genes with similar expressions will be close to each other in the SOM, but there will be no space between groups of genes as in the case of PCA. This makes it hard to assign new groups and large SOMs are therefore more suitable to validate previous classifications based on PCA and hierarchical clustering. The alternative is to use a small SOM, with a rather small number of neurons. This forces the genes to cluster in the few neurons available, thereby creating groups. Testing different parameters for the generation of SOMs and classification of the autoscaled catenated *wild-type*, *HXT-HXT7 *and HXT-TM6* data, we found that a 3 × 2 SOM gave highly reproducible groupings, independent of the learning rate and the number of neighbors. Figure 7 shows the groups formed in such SOM based on a learning rate of 0.10, 2 neighbors and 10000 iterations. The groups agree very well with the PCA classification. In cell (2, 1) we find *CYC1*, *ADH5 *from HXT-TM6* and *HXT-HXT7*, *ADH3 *from HXT-TM6* and *wild-typ*e *SUC2*. These are the genes found in the bottom left of the PC1 vs. PC2 loadings scatter plot (Figure 5). Cell (2, 2) contains only *PGK1 *in HXT-TM6*, which was concluded above to have aberrant expression profile both by PCA and hierarchical clustering. In cell (1, 3) we find the glucose induced genes and *ADH4 *in HXT-TM6* that in the loadings scatter plot are found at negative PC1 and a PC2 around zero. Cell (1, 1) contains most of the glucose-repressed genes, *HSP12 *in HXT-TM6* and *HXT-HXT7*, *ADH3 *in *HXT7 *and *wild-type*, and *ADH5 *in *wild-type*, which in the PCA loadings scatter plot are found at positive PC1, and slightly positive PC2. Cell (1, 2) contains *HSP12 *from *wild-type*, *SUC2 *from HXT-TM6* and *ADH4 *from *HXT-HXT7*. In the PCA loadings scatter plot, HXT-TM6* SUC2 and *wild-type HSP12 *are also close to each other at positive PC2 and a PC1 around zero. *ADH4 *in *HXT-HXT7 *is also in this region, although it is closer to another group of genes that in the SOM is found in unit (2, 3). These are *ADH6 *in all three strains, *ADH4 *in *wild-type*, and *MIG1 *and *PGK1 *in *HXT-HXT7*. The same genes are found in the top left corner of the PC1 vs. PC2 loadings scatter plot.

Classifications by PCA, hierarchical clustering, and SOM are based on quite different assumptions and mathematical models, which makes the methods complementary. Hierarchical clustering and SOM create groups based on some measure of similarity between the samples, which is calculated directly from the experimental data. Additional criteria are required to construct groups (hierarchical clustering) or define regions for groups (SOMs). In contrast, PCA is based on the variables and its main objective is to reveal patterns by calculating a set of abstract factors (the PCs). The number of PCs is much lower than the number of variables, which makes interpretation easier. It eliminates most of the random variation but also some systematic, biologically relevant, information may be missed. SOM takes account of all variation in the data, but at the expense of not having a linear scale. This makes SOM more suitable for validation than first hand classification. Hierarchical clustering creates clusters sequentially by inspecting subpopulations of the data. Once a sample is entered into a group it cannot be extracted again (i.e. hierarchical). Hence, even though all information in the measurement is considered, it is not considered all at once. Therefore, the final clusters may depend on subtle differences between samples' expression profiles. For this reason results of hierarchical clustering should be confirmed by an independent method. For the data presented here and also for the independent replicated experiment in Additional data 3, classifications by PCA, hierarchical clustering and SOM are highly consistent, suggesting that the conclusions reached based on the results are valid.

## Conclusion

**Figure 8:**
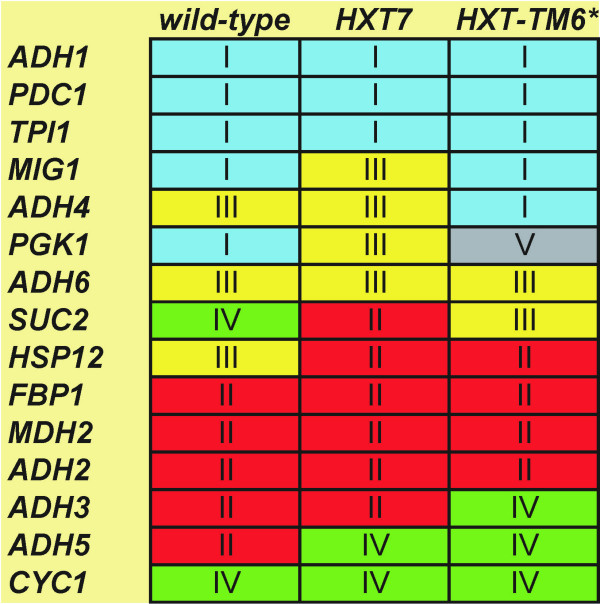
**Summary of gene classification. **Genes are arranged into five groups after PCA: group I (genes induced upon glucose addition), group II (genes repressed upon glucose addition), group III (genes with expression profiles that passes through a maximum), group IV (genes with expression profiles that passes through a minimum) and group V (no regulation). PCA classification was confirmed by hierarchical clustering and Kohonen SOM.

The yeast multiway expression profiles, based on the expression of 15 genes of interest measured at eight time points in fours strains, analyzed catenated by the three analytical methods Principal component analysis, hierarchical clustering and self-organized maps yield highly consistent results. The genes can be divided into four groups (Figure 8) characterized by different combinations of the two main loading vectors of the PCA, they form four hierarchical clusters, and they separate in a SOM with small number of neurons. *PGK1 *in HXT-TM6* strain falls outside these groups in all analyses, indicating it has a distinct expression profile. A further noteworthy observation is that the responses of *MIG1*, *PGK1 *and *SUC2*, in agreement with our previous study [3], depend on the glycolytic rate. The previously rather unknown *ADH3-6 *genes and also *HSP12*, respond to glucose stimuli. *HSP12 *is strongly repressed also in a strain depleted of all glucose transporters, which suggests that Hsp12 senses extracellular glucose. *ADH4 *and *ADH6 *are transiently stimulated by glucose, although neither has been reported to participate in ethanol production [12,35]. *ADH3 *and *ADH5 *are repressed in a seemingly glycolytic rate dependent manor (Figure 8). The detailed temporal expression profiles are quite different in the different strains but our results suggest that there is a fine-tuning regulatory mechanism for the *ADH3-6 *genes that involves glucose. Finally, as the regulation of the *ADH1*, *ADH2 *and *PDC1 *genes remain the same in all strains capable of glucose metabolism our study confirms that the differences in ethanol production between the strains is not regulated at the ethanol production branch.

## Methods

### Strains and growth conditions

Strains used in this study were KOY.2-1C83 (*MATa MAL2-8c SUC2*), KOY.VW100P (MATa *MAL2-8c SUC2 hxt17Δ ura3-52 gal2Δ::loxP stl1Δ::loxP agt1Δ::loxP ydl247wΔ::loxP yjr160cΔ::loxP hxt12Δ::loxP hxt15Δ::loxP hxt16Δ::loxP hxt14Δ::loxP hxt12Δ::loxP hxt9Δ::loxP hxt11Δ::loxP hxt10Δ::loxP hxt8Δ::loxP hxt514:loxP hxt2Δ::loxP hxt367Δ::loxP *carrying an integration cassette at the former *HXT367 *site containing the truncated, constitutive promoter of *HXT7 *[41] (the KlURA3 open reading frame for counter selection, and the *HXT7 *terminator), KOY.HXT7P (same as KOY.VW100P but klURA3, in the integration cassette, was replaced by *HXT7 *and *ura3-52::URA3*) and KOY.TM6*P (same as KOY.VW100P but klURA3 in the integration cassette was replaced by TM6* and *ura3-52::URA3*) (3-4). Detailed strain information has been reported previously [3,5,6]. Cells were grown in 1% ethanol and 5× concentration of minimal media [42] until an optical density (OD) of 1–1.5 at 610 nm. Glucose was pulsed to a final concentration of 5% (w/v) and samples for RNA extraction were taken at 0 min just before the glucose pulse and then at 1, 5, 10, 20, 30 and 60 min after the pulse.

### RNA extraction, cDNA synthesis and quantitative real-time PCR

RNA was extracted using phenol/chloroform extraction. RNA was dissolved in water and 100 μg was DNase treated on RNeasy columns (QIAGEN) as described by the manufacturer. 1 μg of the DNA free RNA was used in the reverse transcriptase reaction (Superscript II, Invitrogen) using pd(T)12–18 (Amersham Bioscience) as primers. Quantitative real-time assays were performed in an iCycler (BIORAD). PCR products were validated by agarose gel electrophoresis and melting curve analysis. Other settings are specified separately in the results. All real-time PCR experiments were run as duplicates. All data analyses, including normalization and data pre-treatment were performed with GenEx (version 4.1.7, MultiD Analyses). Data Supplement 1 and 3 require GenEx for visual inspection of the data.

## Authors' contributions

AS, KE and MK conceived the study. AS and KE performed the experiments, while AS, JMA, BS, AF and MK did the statistical analysis. AS, KE, JMA and MK wrote the manuscript. All authors read and approved the final manuscript.

## Supplementary Material

Additional file 1 Gene expression profiles for *HXT-HXT7*, HXT-TM6* and *HXT-null*. Click here for file

Additional file 2 Matrix-augmented PCA for *wild-type*, *HXT-HXT7*, HXT-TM6* and *HXT-null*. Click here for file

Additional file 3 Matrix-augmented PCA for *wild-type*, *HXT-HXT7 *and HXT-TM6* for an additional data set. Click here for file
