# Supplementary material for: Multiway real-time PCR gene expression profiling in yeast Saccharomyces cerevisiae reveals altered transcriptional response of ADH-genes to glucose stimuli
Source: BMC Genomics. 2008 Apr 16;9:170. doi: 10.1186/1471-2164-9-170 (PMC2335116; doi:10.1186/1471-2164-9-170)
Supplement: Additional file 1 — Gene expression profiles for HXT-HXT7, HXT-TM6* and HXT-null. [file 1471-2164-9-170-S1.pdf]

Additional data file 1

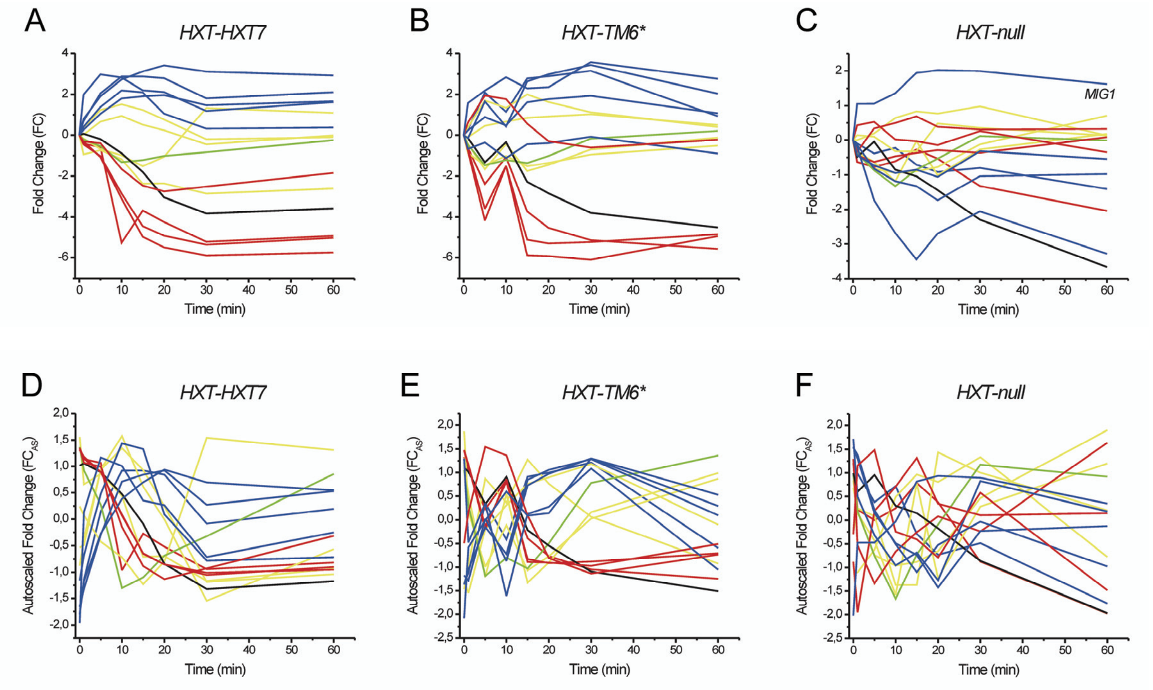

Figure 1. Gene expression profile. The FC (A, B and C) and FC_{AS} (D, E and F) over time are shown for *HXT-HXT7* (A and D), *HXT-TM6** (B and E) and *HXT-null* (C and F) yeast. Note that *MIG1* is the only gene in *HXT-null* that is clearly induced. The following colors are used: Glucose-induced genes (blue), glucose-repressed genes (red), *ADH3-6* (yellow), *HSP12* (black) and *CYC1* (green).
