# Supplementary material for: Multiway real-time PCR gene expression profiling in yeast Saccharomyces cerevisiae reveals altered transcriptional response of ADH-genes to glucose stimuli
Source: BMC Genomics. 2008 Apr 16;9:170. doi: 10.1186/1471-2164-9-170 (PMC2335116; doi:10.1186/1471-2164-9-170)
Supplement: Additional file 2 — Matrix-augmented PCA for wild-type, HXT-HXT7, HXT-TM6* and HXT-null. [file 1471-2164-9-170-S2.pdf]

Additional data file 2

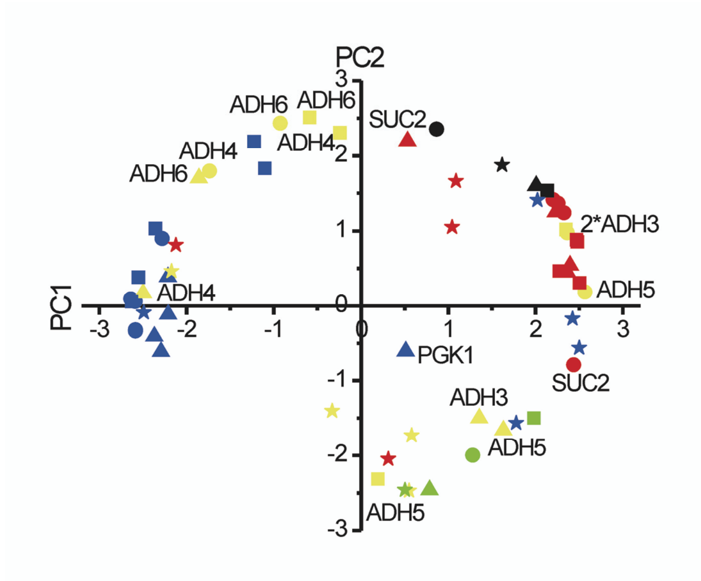

Figure 1. Matrix-augmented PCA for *wild-type*, *HXT-HXT7*, *HXT-TM6** and *HXT-null*. Data matrices for respectively strain were catenated to a single matrices followed by PCA. The following colors and symbols are used: Glucose-induced genes (blue), glucose-repressed genes (red), *ADH3-6* (yellow), *HSP12* (black), *CYC1* (green), *wild-type* (circles), *HXT-HXT7* (squares), *HXT-TM6** (triangles) and *HXT-null* (stars).
