# Supplementary material for: Multiway real-time PCR gene expression profiling in yeast Saccharomyces cerevisiae reveals altered transcriptional response of ADH-genes to glucose stimuli
Source: BMC Genomics. 2008 Apr 16;9:170. doi: 10.1186/1471-2164-9-170 (PMC2335116; doi:10.1186/1471-2164-9-170)
Supplement: Additional file 3 — Matrix-augmented PCA for wild-type, HXT-HXT7 and HXT-TM6* for an additional data set. [file 1471-2164-9-170-S3.pdf]

Additional data file 3

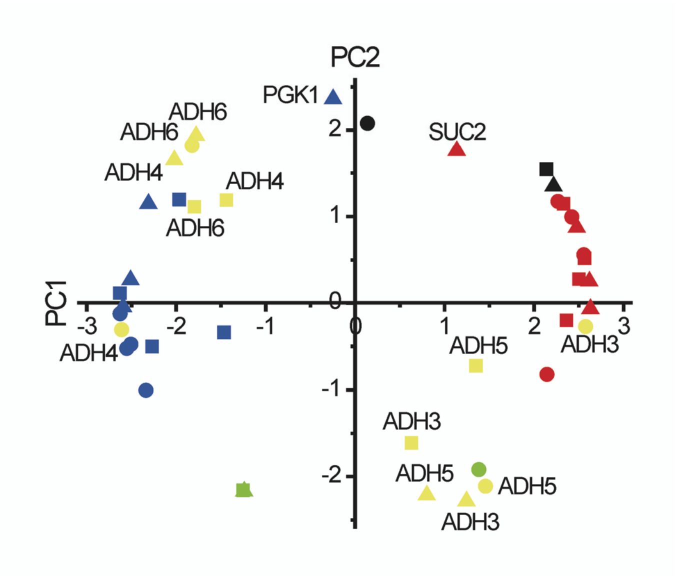

Figure 1. Matrix-augmented PCA for *wild-type*, *HXT-HXT7* and *HXT-TM6** of an independent data set. Data matrices for the strains were catenated into a single matrix, which was analyzed by PCA. The observed gene groups are similar to those in the first data set shown in Figure 5. *MIG1* was not included in this study. The following colors and symbols are used: Glucose-induced genes (blue), glucose-repressed genes (red), *ADH3-6* (yellow), *HSP12* (black), *CYC1* (green), *wild-type* (circles), *HXT-HXT7* (squares) and *HXT-TM6** (triangles).
